# Validation of symptom clusters in patients with metastatic bone pain

**DOI:** 10.3747/co.v15i5.289

**Published:** 2008-10

**Authors:** S. Hadi, L. Zhang, A. Hird, E. de Sa, E. Chow

**Affiliations:** * Rapid Response Radiotherapy Program, Department of Radiation Oncology, Odette Cancer Centre, University of Toronto, Toronto, ON

**Keywords:** Bone metastases, metastatic bone pain, palliative radio-therapy, symptom cluster, symptom cluster instability

## Abstract

**Purpose:**

Symptom clusters (scs) are a dynamic construct. They consist of at least 2 or 3 interrelated symptoms that may be a significant predictor of patient morbidity. In a previous study, we identified 2 scs in patients with bone metastases:

- An activity-related interference cluster
- A psychology-related interference cluster

These scs may be clinically important in the pain and symptom management of patients with metastatic bone pain. It is therefore important to validate the reported scs to determine if they hold true across similar patient populations.

**Patients and Methods:**

From February to September 2007, our study accrued 52 patients with bone metastases [29 men (56%), 23 women (44%); median age: 68.5 years (range: 39–87 years)] who were referred for palliative radiotherapy (rt). Prostate (31%), breast (29%), and lung (19%) were the most common primary cancer sites. Treatment arms ranged from single to multiple fractions, with most patients receiving a single 8-Gy fraction (77%) or 20 Gy in 5 fractions (21%). The most prevalent sites for rt were spine (42%), hips (17%), and pelvis (14%). Worst pain at the site of rt and functional interference scores were assessed using the Brief Pain Inventory (bpi), a multidimensional pain instrument that uses 11-point numeric rating scales. Patients provided their symptom severity scores on the bpi at baseline and at 4, 8, and 12 weeks post rt. At all time points, a principal component analysis with varimax rotation was performed on 8 items (worst pain and 7 functional interference items) to determine relationships between symptoms before and after rt for bone pain.

**Results:**

Two scs were identified. Cluster 1 included worst pain and interference with general activity, normal work, and walking ability; cluster 2 consisted of interference with mood, sleep, enjoyment of life, and relations with others. Our statistical analysis produced varied results for the 2 clusters found in our previous investigation. These differences may be an indicator for the instability of scs or may be a result of the fewer number of patients accrued in the present validation study.

**Conclusions:**

The scs in our two studies were not identical for patients receiving palliative rt for symptomatic bone metastases. Another sc validation study should be conducted with a larger sample before a conclusion is drawn about the existence of an unstable phenomenon in sc research.

## 1. INTRODUCTION

Bone metastasis is a frequent complication of cancer and has been found in 70%–85% of cancer patients at autopsy 1. Metastasis most often occurs in patients with primary breast, prostate, and lung tumours 2. Approximately 50%–75% of patients will require treatment for their metastatic bone pain with the aim of symptom palliation 3. Radiation therapy has been shown to relieve bone pain in approximately 80% of patients 4.

The Brief Pain Inventory (bpi) is a multidimensional instrument that was originally developed in 1994 by Cleeland and Ryan 5 to address the problem of inadequate pain control in cancer patients. The bpi is the most frequently used multiple-item measure of pain in cancer research 6, and it is measured on the sensory and affective dimensions. The sensory component of pain intensity measures worst, average, and current pain, and the affective dimension of functional interference includes general activity, normal work (including work outside the home and housework), walking ability, mood, sleep, relations with others, and enjoyment of life. Previous research found that changes in worst pain are significantly correlated with 6 of the 7 life functions, “relations with others” being the exception 7,8.

The term “symptom cluster” was first coined by Dodd, Miaskowski, and Paul in 2001 in their work with pain, fatigue, and sleep disturbances 9. Symptom clusters have been proposed to consist of at least 2 10 or 3 9 interrelated symptoms in a stable group that is relatively independent of other clusters and that possibly reveals specific underlying dimensions or mechanisms 10. Symptoms within a cluster may or may not have the same underlying cause 9. However, to be considered clustered, symptoms must have a stronger relationship with symptoms in the same cluster than with symptoms in other clusters 10. Because the bpi assesses pain on several dimensions, the present study defined a symptom cluster as 2 or more interrelated symptoms or functional interference items.

From May 2003 to January 2007, we conducted a previous study at the Odette Cancer Centre and, using the bpi, extracted 2 symptom clusters in patients receiving palliative radiation therapy (rt) for symptomatic bone pain 11. The 348 individuals who agreed to participate in the study completed the bpi before rt (baseline) and at weeks 4, 8, and 12 post rt 11.

Two symptom clusters were identified at baseline:

- An activity-related interference cluster (cluster 1)
- A psychological-related interference cluster (cluster 2)

Cluster 1 consisted of worst pain and interference with normal work, general activity, walking ability, and enjoyment of life. Cluster 2 consisted of interference with relations with others, sleep, and mood. In responders to rt, no symptom clusters were identified in the follow-up assessments. However, in non-responders to radiation, symptom clusters appeared at week 8 post rt. Symptom clusters appear to be unstable, and so it is clinically important to validate reported symptom clusters found in previous research to determine if they hold true across similar patient populations.

The primary objective of the present study was to validate the findings from our previous study 11 by comparing the extracted symptom clusters at baseline and at 4, 8, and 12 weeks post rt.

## 2. PATIENTS AND METHODS

The Rapid Response Radiotherapy Program (rrrp) at the Odette Cancer Centre is an innovative program that was initiated in 1996 to provide timely palliative rt for symptom relief in patients with advanced disease 12. The rrrp provides an opportunity for cancer patients to be assessed, planned, and treated on the day of first consultation so as to relieve symptomatic cancer pain and to maintain or improve quality of life.

All patients referred to the rrrp for palliative rt of symptomatic bone metastases were considered for this study. For study participation, patients had to be at least 18 years of age, to have radiologic evidence of bone metastases, and to provide informed consent. Patients were excluded if there was a language barrier or if they had experienced a pathologic fracture or spinal cord compression.

From February to September 2007, 52 patients from the rrrp were enrolled into the study. At initial consultation, patients with bone metastases were asked to rate their worst pain and functional interference scores on the bpi using 11-point numeric rating scales. The numeric rating scales had descriptive anchors of 0 for “no pain” or “does not interfere” and 10 for “worst imaginable pain” or “completely interferes.”

All reference to pain was specific to the irradiated site in these patients. Patient demographics, which included age, sex, cancer history, Karnofsky performance status ( ) 13 kps , and analgesic consumption during the preceding 24 hours were recorded at the first visit. Opioid analgesics were converted to total daily oral morphine equivalent doses. The progress of a patient’s response to palliative rt was monitored using the bpi at 4, 8, and 12 weeks post rt. A research assistant was responsible for obtaining bpi scores in telephone interviews.

Patient confidentiality was maintained, and patients were assigned a unique number for study identification purposes. Ethical approval was obtained from the hospital research ethics board, and all questionnaire administration and information collection was performed by a trained research assistant. The entire process was consistent with the principles set out in the Declaration of Helsinki on conducting clinical research.

Our study defined responders to radiation treatment as patients experiencing a complete (cr) or partial response (pr). The International Bone Metastases Consensus Working Party 14 defines “complete response” as a pain score of 0 at the irradiated site, with no concomitant increase in analgesic intake (stable or reduced analgesics in daily oral morphine equivalent doses). It defines “partial response” as a pain reduction of 2 points or more at the irradiated site on a 0–10 scale without analgesic increase or with an analgesic reduction of 25% or more from baseline without an increase in pain 14.

### 2.1 Statistical Analyses

Results are expressed as mean ± standard deviation or median and range for quantitative variables, and as proportions for categorical findings. The chi-square test was used to test for differences in the averages of symptom severity and of functional interference scores between the sexes. The Spearman correlation was applied at baseline to determine the strength of the correlation between any 2 of the 8 items.

Principal component analysis (pca) with varimax rotation was applied to worst pain and the 7 functional interference items. To determine relationships between items before and after rt for bone pain, pca was performed on the 8 items at each time point for responders and for non-responders alike.

The highest factor loading score predicted the assignment of individual symptoms to an independent factor. The Cronbach alpha statistic was used to estimate the internal consistency and reliability of the derived clusters at baseline and at subsequent follow-ups. Using a biplot graphic, robust relationships and correlations between the 8 items were displayed; the length and proximity of arrows acted as determinants of the strength of the correlations. The final communality refers to the percentage variance in an observed variable that was accounted for by the retained clusters.

A general linear mixed model was used to determine whether the bpi items changed over time (from baseline to week 12) in all patients and whether a responder effect occurred over time (from week 4 to week 12). Results were considered significant at the 5% critical level (*p* < 0.05). All calculations were performed using the sas (version 9.1: SAS Institute, Cary, NC, U.S.A.) and s-plus (version 7.0: Mathsoft, Cambridge, MA, U.S.A.) statistical software packages.

## 3. RESULTS

From February to September 2007, our study accrued 52 patients with symptomatic bone metastases [29 men (55.8%), 23 women (44.2%); median age: 68.5 years (range: 39–87 years)] who received rt. All participants provided complete baseline data at initial consultation. Table I summarizes patient demographics and disease information. These patients had a median kps of 70 (range: 40–90) and a median daily morphine equivalent dose of 10 mg. Prostate (30.8%), breast (28.9%), and lung (19.2%) were the most common primary cancer sites. Most patients received a single fraction of 8 Gy to the spine.

Table II lists the prevalences, sex differences, and median severities for “worst pain” and the 7 functional interference items. A symptom was considered present if it was scored greater than 0. All 52 patients had a “worst pain” score.

The 3 most prevalent interference items were general activity (88.2%), enjoyment of life (88.0%), and normal work (85.7%). For individuals who were experiencing interference, all 8 items had a moderate-to-severe functional median severity score, with normal work being the highest (median score: 8). In using chi-square analyses to test for sex differences, we observed no significant difference between women and men on any bpi item except for relations with others (*p* = 0.029).

Before rt, some of the patients did not experience certain functional interference items. For those who provided a score, Table III lists the symptom distress of patient-assessed worst pain and of functional interference scores. The median symptom distress ranged from 3.5 to 7 for the 8 items. “Worst pain” and “normal work” had the highest symptom distress, with “relations with others” ranking lowest.

The Spearman correlations in Table IV depict the strength of the relationships between the 8 items. Each Spearman correlation was highly significant (*p* < 0.0001), except for the relationships of sleep with pain (*p* = 0.0008), with general activity (*p* = 0.0004), with walking ability (*p* = 0.0068), and with relations with others (*p* = 0.0029). Other nonsignificant Spearman correlations were observed for enjoyment of life with walking ability (*p* = 0.0014) and with normal work (*p* = 0.0003), and for relations with others with normal work (*p* = 0.0015). Correlations between items ranged from 0.38 to 0.88, with the lowest correlation occurring between sleep and walking ability, and the highest correlation occurring between normal work and general activity.

The pca with varimax rotation extracted principal components with a minimum Eigenvalue of 0.75 that each explained more than 10% of the total variance. Two components or symptom clusters were extracted from the questionnaire completed before rt, accounting for 77% of the total variance.

**FIGURE 1:**
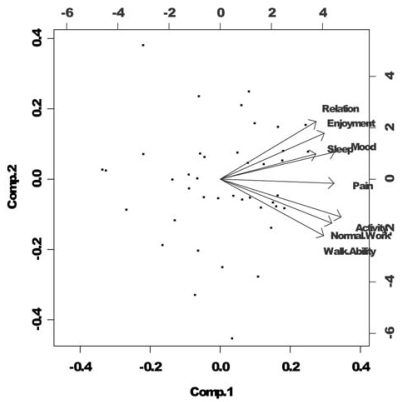
Biplot between components 1 and 2.

Cluster 1 included walking ability, general activity, normal work, and worst pain; it accounted for 67% of the total variance. Cluster 2 included relations with others, enjoyment of life, mood, and sleep; it accounted for 10% of the total variance. Using the Cronbach alpha, the internal reliabilities of the two clusters were high at 0.85 in cluster 2 and 0.92 in cluster 1, demonstrating good internal consistency (Table V). The final communalities showed that all the variables were well accounted for by the two clusters, with final communality estimates ranging from 0.57 for sleep to 0.93 for general activity. Figure 1 shows a biplot for the two principal components, cluster 1 and cluster 2, depicting a two-dimensional model. The two clusters are distinct, being that they have different orientations. The arrows of longer length and closer proximity suggest a higher correlation between symptoms.

Because of attrition, follow-up response rates at weeks 4, 8, and 12 were respectively 41.2%, 35.0%, and 23.8% of the 52 patients. Using a general linear mixed model, all worst pain and functional interference scores significantly decreased over time (*p* < 0.0001), except for mood (*p* = 0.0002) and relations with others (*p* = 0.0047, Table VI). However, total oral morphine equivalent dose did not display a significant change from baseline to week 12 (*p* = 0.8091, Table VI).

The percentages of patients who responded to rt throughout the follow-up assessments were 60.1% at week 4, 67.9% at week 8, and 73.7% at week 12. Table VII sets out the number of patients at each time point and the proportion that had a cr or pr to rt.

Because of the small sample size in the responder and non-responder groups alike, we decided not to run the pca to extract symptom clusters. Any symptom clusters extracted would not be reliable from a statistical viewpoint. Table VIII summarizes the symptom cluster dynamics throughout the duration of the study. In keeping with the criteria of extracting symptom clusters with a minimum Eigenvalue of 0.75 and a minimum proportion of variance of 10%, 2 clusters could be extracted at baseline, at week 4, and at week 8. However, these clusters did not remain consistent over time; they varied at each time point. The functional interference items that clustered consistently were walking ability and general activity in cluster 1 and relations with others and mood in cluster 2. The pair of items in cluster 2 clustered together with those found in cluster 1 after the initiation of rt in weeks 4 and 8. At week 12, no symptom clusters that met the symptom cluster criteria could be identified.

## 4. DISCUSSION

Our findings on clusters match the two factors extracted in a Norwegian validation study of the bpi 15. Klepstad and colleagues performed a principal factor analysis with direct oblimin solution on a sample of 300 hospitalized cancer patients [55% men; median age: 63 years; median kps: 70 (range: 10–90)] 15. The most common primary cancer sites were breast (20%), prostate (20%), and lung (18%). Confirmed metastatic disease was diagnosed in 235 patients. Klepstad *et al.* were able to extract 2 factors: interference with physical function (general activity, walking ability, and normal work), and interference with psychological function (mood, relations with others, sleep, and enjoyment of life) 15. A third factor of pain severity was extracted in the Norwegian study, which included worst, least, average, and current pain. However, only worst pain was included in our study’s analysis. Consequently, worst pain compounded the activity-related interference found in our initial symptom cluster research in cancer patients with metastatic bone pain 11.

The findings in the present validation study differ slightly from our initial symptom cluster research 11. We extracted 2 symptom clusters before rt start, calling cluster 1 “activity-related interference” and cluster 2 “psychology-related interference.” These clusters were reproducible at baseline in the current validation study; however, in the previous study, enjoyment of life was more highly correlated with the activity-related interference items and compounded cluster 1 (activity-related interference) 11.

The initial findings in our previous study showed that the statistical analysis failed to extract any symptom clusters in the responder group after initiation of rt^11^. The symptom clusters had dispersed at weeks 4, 8, and 12, and no cluster could be extracted with further statistical analysis (that is, pca). The disintegration of the symptom clusters was attributed to the alleviation of symptomatic bone pain, which thus had a direct influence on the functional interference in all 7 items. The dynamics of the symptom clusters in the responders showed that all items were affected, thus further supporting the validity of the bpi 5.

The current validation study was able to extract 2 symptom clusters at weeks 4 and 8, and yet it failed to extract any symptom clusters in week 12 of the follow-up assessments. Although 2 symptom clusters were observed, these clusters varied at each time point. The items rearranged themselves to cluster with different items in weeks 4 and 8.

Among the 8 items, walking ability and general activity consistently remained together in cluster 1 (activity-related interference). The consistency for this pair of items is evidence of a common underlying construct, which supports the theory developed by Kim *et al.* in 2005 10. (Walking ability and general activity both involve considerable physical activity of the lower limbs.) On the other hand, relations with others and mood consistently remained together, yet transferred from cluster 2 (psychology-related interference) at baseline to cluster 1 (activity-related interference) in weeks 4 and 8. The influence of pain on psychological symptoms may be related to a combination of physical suffering and a patient’s interpretation of pain in the context of malignant disease.

At baseline, the 2 symptom clusters of activity-related and psychology-related interference in the present study differ slightly from those seen in the previous symptom cluster study 11. In the present research, enjoyment of life had a stronger correlation with psychology-related interference items than with activity-related interference items (as in the past). We also observed that “worst pain” had a higher correlation with walking ability and general activity in cluster 1 and therefore remained with these items at week 4. Interestingly, “worst pain” clustered out of cluster 1 (activity-related interference) at week 4 and showed a stronger correlation with interference with sleep in cluster 2 at week 8 (Table VIII). We are unable to explain why a patient’s worst pain would cluster with a subdued or inactive state of sleeping than with a state that requires vigorous activity and stress on a patient’s body, such as walking ability, general activity, and normal work.

Symptom clusters are a dynamic construct and remain unpredictable across varied treatments, conditions, and time periods. This symptom cluster phenomenon is not limited to the present research, but has also been observed in earlier studies with symptom clusters 16,17. Using the Edmonton Symptom Assessment Scale on patients with bone 16 and brain metastases 17, these two studies were able to extract symptom clusters at baseline that rearranged at subsequent follow-up weeks. Certain clusters changed after rt; others remained stable. As a result, the findings in our present study are not unexpected.

Kirkova and Walsh 18 refined the term “cluster stability” in an editorial published in *Supportive Care in Cancer.* They defined cluster stability as a cluster composition across subjects and time. It can be conceptualized as specific clusters that exist in a variety of patient populations or those influenced by a common intervention.

Symptom clusters are a dynamic construct and are influenced by a specific symptom, its severity, treatment, primary cancer site, stage of disease, and symptom meaning 19–21. Bone metastases may have a different meaning to the patient at different times during the disease trajectory 18. This difference may explain the variation of symptom clusters in the present study from baseline to weeks 4 and 8.

In comparing the present validation study to the previous study conducted in 2007, we observed that patients in the 2007 group had higher median “worst pain” and functional interference (specifically, general activity, walking ability, and enjoyment of life) scores. They had less interference with relations with others. Median scores for mood and sleep interference were identical in both studies. The severity of an individual’s bone pain symptoms may determine greater cluster variability 18. Our study did not test for significance, but the differences in median score may account for the cluster instability across the two studies.

### 4.1 Limitations

The differences between the two studies may be attributable to the lesser number of patients in the present validation study. A sample size of 52 patients is significantly less than a population of 348 patients.

Comparisons between symptom cluster studies remain difficult. The variations in cluster composition are a direct result of the measurement instrument, the study methodology, the statistical analysis, and the cutoff points used to determine a symptom cluster 22–24. Comparisons also remain an obstacle because no consensus has yet been reached concerning the definition of a symptom cluster and the clinical meaning of a derived cluster.

The present validation study adds to the exploration and further understanding of symptom cluster dynamics and the possible instability of symptom clusters over time and over subjects. Symptom cluster research is still being refined, but it may eventually facilitate diagnosis and treatment follow-up, and may aid in predicting survival in clinical practice 18.

An additional validation study with a larger patient population is highly recommended. The sample size in the present study was not large enough to produce reliable results. To rule out the phenomenon of cluster instability, more studies must be conducted using the same methodology of extracting symptom clusters: for example, use of a standard instrument; similar patient populations; identical definitions, criteria, and cut-off points; and repeated assessments at appropriate time intervals.

## 5. CONCLUSIONS

The refinement of definitions, development of criteria, and determination of optimal statistical methods could help to develop symptom cluster research standards and, hopefully, to identify clinically important clusters 18.

Following verification of a symptom cluster, subsequent research has to determine whether that cluster occurs in other patient samples and whether it constitutes a “symptom cluster diagnosis” 18. The same study should be repeated in patient samples other than a bone metastases group to see whether similar symptom clusters can be extracted on the basis of cancer-related pain in general.

## Figures and Tables

**TABLE I:** Patient characteristics

Characteristic	Value
Patients (*n*)	52
Sex [*n* (%)]	
Male	29 (55.8)
Female	23 (44.2)
Age at radiation (years)	
Mean ± SD	66.9±11.6
Median (range)	68.5 (39–87)
Karnofsky performance status	
Mean ± SD	70.2±12.8
Median (range)	70 (40–90)
Total morphine equivalent (mg)	
Mean ± SD	77.6±163
Median (range)	10 (0–880)
Primary cancer sites [*n* (%)]	
Prostate	16 (30.8)
Breast	15 (28.9)
Lung	10 (19.2)
Bladder	4 (7.7)
Pancreas/gastric	3 (5.8)
Others	4 (7.7)
Sites of radiotherapy [*n* (%)]	
Spine	22 (42.3)
Hips	9 (17.3)
Pelvis	7 (13.5)
Shoulders	5 (9.6)
Rib or ribs	4 (7.7)
Extremities	2 (3.8)
Other	3 (5.8)
Radiation dose [*n* (%)]	
800 cGy/1 fraction	40 (76.9)
2000 cGy/5 fractions	11 (21.2)
3000 cGy/10 fractions	1 (1.9)

SD = standard deviation.

**TABLE II:** Prevalence, sex difference, and median severity of worst pain and 7 functional interference items

Symptom	Functional prevalence					Functional severity a
	Total *(*n*)*	Overall *[*n *(%)]*	Men *[*n *(%)]*	Women *[*n *(%)]*	χ^2^ p *Value*^b^	Median *(range)*
Worst pain	52	52 (100)	29 (100)	23 (100)	0.999	7 (1–10)
General activity	51	45 (88.2)	24 (85.7)	21 (91.3)	0.523	7 (2–10)
Enjoyment of life	50	44 (88.0)	25 (92.6)	19 (82.6)	0.279	7 (1–10)
Normal work	42	36 (85.7)	18 (85.7)	18 (85.7)	0.999	8 (2–10)
Walking ability	50	40 (80.0)	21 (77.8)	19 (82.6)	0.670	7 (2–10)
Mood	51	40 (78.4)	22 (78.6)	18 (78.3)	0.979	7 (1–10)
Sleep	51	40 (78.4)	20 (71.4)	20 (87.0)	0.180	6 (1–10)
Relations with others	50	31 (62.0)	13 (48.2)	18 (78.3)	0.029	6 (1–10)

a Functional severity in patients who scored greater than zero. 1 = minimum functional severity; 10 = worst possible functional severity.

b Sex difference as compared by chi-square test. “Total” is all patients who scored the item. “Overall” is all patients who scored the item as greater than 0.

**TABLE III:** Worst pain scores and functional interference items at baseline (scale: 0–10)

Symptom	Patients (n)	Mean *± SD*	Median (range)
Worst pain	52	6.50±2.57	7 (1–10)
Normal work	42	6.24±3.46	7 (0–10)
General activity	51	5.90±3.20	6 (0–10)
Enjoyment of life	50	5.74±2.96	6 (0–10)
Walking ability	50	5.62±3.58	6 (0–10)
Mood	51	4.92±3.58	5 (0–10)
Sleep	51	4.90±3.61	5 (0–10)
Relations with others	50	3.60±3.48	3.5 (0–10)

SD = standard deviation.

**TABLE IV:** Spearman correlation among worst pain and functional interference items at baseline

Items	Worst pain	General activity	Mood	Walking ability	Normal work	Relations with others	Sleep	Enjoyment of life
Worst pain	1							
General activity	0.65	1						
Mood	0.66	0.77	1					
Walking ability	0.56	0.80	0.60	1				
Normal work	0.61	0.88	0.67	0.72	1			
Relations with others	0.58	0.62	0.70	0.58	0.48	1		
Sleep	0.45	0.48	0.57	0.38	0.57	0.41	1	
Enjoyment of life	0.59	0.59	0.62	0.44	0.53	0.58	0.55	1

a *p* Value of Spearman correlation is highly significant (<0.0001) between items, except for sleep with worst pain (*p* = 0.0008), with general activity (*p* = 0.0004), with walking ability (*p* = 0.0068), and with relations with others (*p* = 0.0029); enjoyment of life with walking ability (*p* = 0.0014) and with normal work (*p* = 0.0003); and relations with others with normal work (*p* = 0.0015).

**TABLE V:** Factor loadings and final communality from the principal component analysis

	Component^a^		
	1	2	Final communality
Walking ability	**0.86**	0.25	0.81
General activity	**0.86**	0.44	0.93
Normal work	**0.84**	0.36	0.84
Worst pain	**0.64**	0.59	0.75
Relations with others	0.21	**0.82**	0.73
Enjoyment of life	0.32	**0.80**	0.75
Mood	0.48	**0.76**	0.80
Sleep	0.39	**0.65**	0.57
Variance (%)	67	10	
Cronbach alpha	0.92	0.85	

a Boldface indicates a relationship between the functional interference items in each component.

**TABLE VI:** Distress scores of worst pain and functional interference items of all patients (Pts) over time a

Item	Baseline		Week 4		Week 8		Week 12	
	Pts (n)	Mean ± SD (median)	Pts (n)	Mean ± SD (median)	Pts (n)	Mean ± SD (median)	Pts (n)	Mean ± SD (median)
Worst pain	52	6.5±2.6 (7)	33	3.7±3.2 (4)	28	2.6±3.3 (0.5)	19	3.1±3.3 (2)
General activity	51	5.9±3.2 (6)	31	3.7±3.8 (3)	27	2.6±3.4 (0)	19	2.7±3.2 (0)
Mood	51	4.9±3.6 (5)	32	3.4±3.8 (1.5)	28	2.0±3.2 (0)	19	2.2±3.0 (0)
Walking ability	50	5.6±3.6 (6)	32	3.5±3.5 (2.5)	28	2.6±3.5 (0)	19	2.8±3.7 (1)
Normal work	42	6.2±3.5 (7)	29	3.9±4.0 (3)	27	2.9±3.9 (0)	19	3.1±3.8 (2)
Relations with others	50	3.6±3.5 (3.5)	32	2.0±3.1 (0)	28	1.8±3.2 (0)	19	1.8±2.6 (0)
Sleep	51	4.9±3.6 (5)	31	2.8±3.1 (1)	27	1.8±3.0 (0)	19	1.8±2.9 (0)
Enjoyment of life	50	5.7±3.0 (6)	32	3.8±3.6 (3.5)	28	2.6±3.4 (0.5)	19	2.6±3.0 (2)
Total morphine equivalent	49	77.6±163.4 (10)	30	88.8±134.3 (3.5)	27	47.9±123.7 (0)	16	54.2±135.0 (0)

a Using a general linear mixed model, all pain and functional item scores decreased significantly over time (*p* < 0.0001), except for mood (*p* = 0.0002) and relations with others (*p* = 0.0047). However, total oral morphine equivalent dose did not present a significant change over time (*p* = 0.8091).

SD = standard deviation.

**TABLE VII:** Response rates

	At 4 weeks	At 8 weeks	At 12 weeks
Patients (*n*)	33	28	19
Complete response a [*n* (%)]	9 (27.3)	12 (42.9)	5 (26.3)
Partial response a [*n* (%)]	11 (33.3)	7 (25.0)	9 (47.4)
Responders [*n* (%)]	20 (60.1)	19 (67.9)	14 (73.7)
Non-responders [*n* (%)]	13 (39.4)	9 (32.1)	5 (26.3)

a Complete and partial responses were defined as set out by the International Consensus of Bone Metastases Consensus Working Party on palliative radiotherapy endpoints 14.

**TABLE VIII:** Summary of symptom cluster changes from baseline to subsequent follow-ups in all patients at each time point

Statistics	Value	Items a
**At baseline**		
Min. Eigenvalue	0.77	
Min. proportion of variance	10%	
Cronbach alpha, cluster 1	0.92	Worst pain, **walking ability, general activity,** normal work
Cronbach alpha, cluster 2	0.85	**Relations with others, mood,** enjoyment of life, sleep
**At week 4**		
Min. Eigenvalue	0.86	
Min. proportion of variance	11%	
Cronbach alpha, cluster 1	0.95	Worst pain, **walking ability, general activity, relations with others, mood**
Cronbach alpha, cluster 2	0.84	Normal work, sleep, enjoyment of life
**At week 8**		
Min. Eigenvalue	0.85	
Min. proportion of variance	11%	
Cronbach alpha, cluster 1	0.96	**Walking ability, general activity,** normal work, **relations with others, mood,** enjoyment of life
Cronbach alpha, cluster 2	0.77	Worst pain, sleep
**At week 12**		
Min. Eigenvalue	0.44	
Min. proportion of variance	5%	
No clusters		

a Boldface indicates the items that consistently clustered together despite the movement between clusters.
